# Characterization of *Borrelia*-Derived Extracellular Vesicles: Implications for Pathogenesis and Diagnostics

**DOI:** 10.3390/microorganisms14030600

**Published:** 2026-03-07

**Authors:** Barbara Birkaya, Ahana Byne, Sumaiya Irfan, Joseph Gallagher, Dominic Granato, Hayat Kharmoud, Andrea Blake Brothers, Elsa Ronzier, Amanda Haymond Still, Weidong Zhou, Robert K. Ernst, Hope McIntyre, Ashley Michelle Groshong, Lance A. Liotta, Alessandra Luchini

**Affiliations:** 1 Center for Applied Proteomics and Molecular Medicine, George Mason University, Manassas, VA 20110, USAhkharmou@gmu.edu (H.K.);; 2 College Dean’s Office, American University, Washington, DC 20016, USA; abrothe@gmu.edu; 3 Biomedical Research Laboratory, Institute for Biohealth Innovation, George Mason University, 10650 Pyramid Place, Manassas, VA 20110, USA; 4 Department of Microbial Pathogenesis, University of Maryland School of Dentistry, Baltimore, MD 21021, USA; 5 Lyme Hope, LLC, Mount Airy, MD 21771, USA; 6 Laboratory of Bacteriology, Rocky Mountain Laboratories, Division of Intramural Research, National Institute of Allergy and Infectious Diseases, National Institutes of Health, Hamilton, MT 59840, USA; ashley.groshong@nih.gov

**Keywords:** *Borrelia*, urine, bacterial extracellular vesicles, microglia, NRF2, phagocytosis, TNF-alpha, peptidoglycan, p66, FlaB, monoclonal antibody, C3H/HeJ mice

## Abstract

The cause of chronic neurological effects associated with Lyme disease (LD) remains unclear. We propose that bacterial extracellular vesicles (BEVs) released by *Borrelia burgdorferi*, the causative agent of LD, exacerbate spirochete-induced damage and serve as a persistent source of antigenic stimulation. We showed that, over a 10-day period, in vitro cultures of *B. burgdorferi* B31 produced 38,000 BEVs per spirochete with a distinctive double-membrane structure and median diameter of 143.3 nm. BEVs contained known immunogenic and immunomodulatory molecules such as peptidoglycan, p66, flagellar filament protein (FlaB), basic membrane proteins A/B/D, BdrV, GroEL, CRASP-1, ErpA8, glycerophosphodiester phosphodiesterase, p37, OMS28, p13, OspA/B/C, VlsE, and outer membrane glycolipids (e.g., cholesteryl 6-O acyl beta D galactopyranoside). Chromosome-encoded *16S ribosomal RNA* and cp32 plasmid-encoded *OspE* and *terminase* genes were also detected in the BEVs. Of the 45 *Borrelia* proteins identified in the urine of a C3H/HeJ murine model of Lyme disease, 14 were associated with BEVs. In human urine samples, 31 of 289 spirochete proteins detected in patients with either acute Lyme disease or persistent *borreliosis* post-treatment symptoms, including p66 and FlaB, were also BEV-associated. BEV treatment of HMC3 human microglial cells reduced phagocytic activity and triggered aberrant activation of inflammatory and immunometabolic pathways, including upregulation of interferon-alpha (IFN-α), aconitate decarboxylase 1 (Acod1), and Toll-like receptor 2 (TLR2) gene expression. BEVs also induced NRF2 nuclear translocation. In conclusion, these findings support that BEVs can amplify spirochete-induced damage and act as antigenic debris, driving dampened phagocytic activity and dysregulated inflammation, with implications for diagnostics and therapeutics targeting vesicle-mediated pathology.

## 1. Introduction

Lyme disease (LD), caused by the spirochete *Borrelia burgdorferi* [1] and transmitted through *Ixodes* tick bites, represents the most prevalent vector-borne illness in the Northern Hemisphere, with an estimated incidence exceeding 400,000 cases annually in the United States [2]. Early manifestations, erythema migrans, fever, and fatigue, typically resolve with antibiotics like doxycycline or amoxicillin. One of the most frequent presentations of early disseminated LD in North America is cranial neuropathy, with a predilection for the facial nerve, which can result in facial palsy [3]. In the late stages, peripheral nervous system (PNS) pain and sensory dysfunction are reported, while the most common chronic neurological involvement is a mild encephalopathy [4,5,6,7,8,9]. To explain the etiology of Lyme-associated long-lasting symptoms, prior studies have proposed persistent antigenic irritation in the synovial joint and characterized associated cytokine profiles [10,11,12,13,14,15,16]. Jutras and colleagues detected *Borrelia* peptidoglycan in Lyme arthritis synovial fluid, where they proposed it acts as an immunogen driving arthritis pathogenesis [10].

In this study, we propose the hypothesis that bacterial extracellular vesicles (BEVs) serve as persistent antigenic debris in the nervous system even in the absence of the pathogen. BEVs are resistant to antibiotics targeting cell division, microenvironment chemicals, and enzymatic degradation [17,18]; they can deliver immunogenic cargo systemically, amplify spirochete-induced damage, and persist locally in the absence of replicating spirochetes.

BEVs have emerged as critical players in host-microbe interactions across diverse species of human interest. In Gram-negative bacteria like *Escherichia coli* and *Pseudomonas aeruginosa*, outer membrane vesicles (OMVs) deliver toxins, adhesins, and nucleic acids, modulating host immunity and promoting bacterial survival [19,20]. Gram-positive bacteria, such as *Staphylococcus aureus*, produce membrane-derived vesicles with similar functions [21]. *B. burgdorferi*, a diderm spirochete with a unique cell envelope lacking lipopolysaccharide but rich in lipoproteins and peptidoglycan, produces BEVs with distinctive characteristics [22,23,24,25]. Early studies reported the release of vesicular structures by *Borrelia* during in vitro cultures and in the tick vector [22,23,24,25]; however, their molecular composition in the mammalian host and their contribution to disease pathophysiology remain largely unexplored. In this study, we propose to use urine for detecting spirochete-derived components. Urine integrates molecular cargo from the systemic circulation over the interval between bladder emptying cycles and has therefore been leveraged to study pathologies not only of the kidney but also of non–urinary tract organs, including the liver and brain [26,27,28]. Microbiome-derived BEVs have been shown to influence a variety of host pathophysiological processes, including breast cancer survival and progression through modulation of calcium handling [29], and are hypothesized to contribute to neuroinflammation during the development of neurodegenerative diseases [30,31].

Microglia, specialized nervous system immune cells [32], orchestrate neuroinflammation in response to pathogens or damage-associated molecular patterns (DAMPs). Microglial dysregulated phagocytic activity exacerbates innate immune responses and is associated with brain tissue damage, as seen in neurodegenerative diseases [33,34]. Gram-negative BEVs activate microglia through Toll-like receptors (TLRs), leading to cytokine release (e.g., IL-6, TNF-α) and metabolic reprogramming mediated by enzymes such as Acod1 [35]. This mechanism has been observed with BEVs from *Helicobacter pylori* [35], gut commensals [36], *Bacteroides thetaiotaomicron* [37], and other Gram-negative bacteria [38]. Acod1, which is also induced by *Borrelia* in macrophages [39], produces itaconate, which modulates inflammation but may lead to immune paralysis if dysregulated [40].

In this study, we present the most comprehensive *B. burgdorferi* BEV characterization to date, addressing their production kinetics, size, morphology, and molecular composition at the protein, DNA, and lipid levels. We show that BEVs contain known immunogens, including p66, FlaB, OspA, and peptidoglycan. Using proteomic mass spectrometry, we demonstrated the ability to detect *Borrelia* proteins in infected mouse urine. Additionally, we detected spirochetal proteins in urine from patients with acute LD and those with persistent symptoms of borreliosis following treatment using proteomic mass spectrometry. Proteins detected in both mouse and human urine overlapped with proteins identified in in vitro-generated BEVs. We demonstrated that *Borrelia* proteins are specifically associated with purified urinary extracellular vesicles (uEVs) using novel antibodies targeting FlaB and p66 sequences conserved across multiple Lyme-associated *Borrelia* species [5]. In cell challenge studies, BEVs reduced the phagocytosis efficiency of HMC3 microglia, induced IFN-α, Acod1, and TLR2 gene overexpression, and NRF2 nuclear translocation, thus activating inflammatory and immunometabolic pathways. These findings support the systemic circulation of BEV markers in the mammalian host and link BEVs to dysregulated inflammatory responses in human microglia, thus highlighting BEVs as potential diagnostic and therapeutic targets.

## 2. Materials and Methods

### 2.1. B. burgdorferi Culture and BEV Isolation

**Figure 1:**
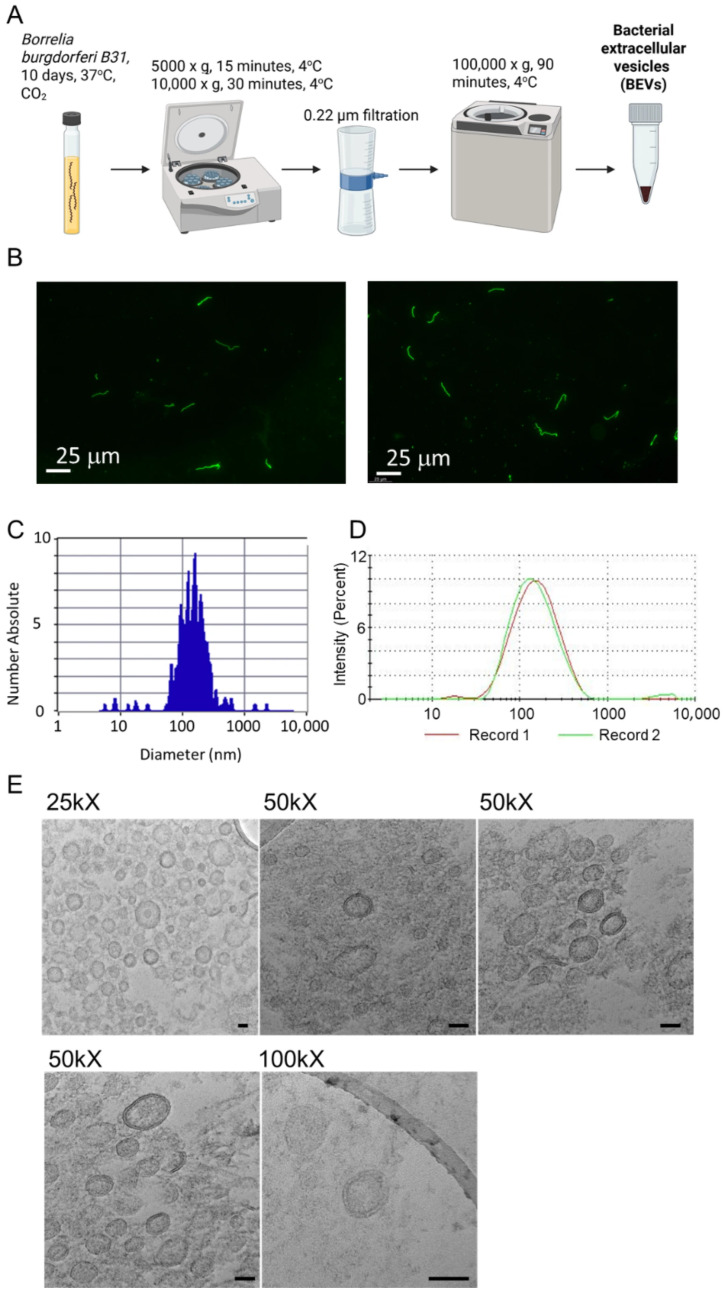
Isolation and characterization of *Borrelia burgdorferi* B31 bacterial extracellular vesicles (BEVs). (**A**) Schematic workflow for BEV isolation from *B. burgdorferi* B31 cultures using sequential centrifugation, 0.22 µm filtration, and ultracentrifugation. (**B**) Fluorescence microscopy images of *B. burgdorferi* B31 stained with FITC-conjugated anti-*Borrelia* antibodies, demonstrating intact spirochete morphology. Scale bar = 25 µm. (**C**) Representative nanoparticle tracking analysis (ZetaView) and (**D**) dynamic light scattering (DLS) measurements of the BEV size distribution. (**E**) Transmission electron microscopy (TEM) images at increasing magnifications (25,000×, 50,000×, and 100,000×) show BEVs as ~100–150 nm circular vesicles with electron-dense borders and visible double-membrane structure. Scale bar = 100 nm.

A *Borrelia burgdorferi* B31 (*Bb*B31) ATCC stock was expanded in bulk culture and frozen in two formats: large (1 mL) and small (250 μL) aliquots. Large aliquots were primarily used to create freezer stabs or to initiate small-volume cultures (1–1.5 mL BSK-II). After incubating for 5–7 days, once the culture reached a countable density, it was scaled up to a larger volume. In contrast, small aliquots were thawed and directly used to inoculate larger batches of BSK-II medium, allowing no more than 10% void volume in tubes. Spirochetes were visualized using FITC-conjugated anti-*Borrelia* antibodies (Abcam, Cambridge, UK). Spirochetes were counted by methanol fixation and trypan blue staining using a chemo-cytometer. Briefly, 100–500 µL of *Bb*B31 culture was spun down at 5000× *g* for 15 min and washed twice with PBS. The pellet was resuspended in 10X pellet volume ice-cold methanol, followed by incubation for 10 min at −20 °C to fix and permeabilize the spirochetes. After incubation, an equal volume of trypan blue dye was added and incubated for another 5 min, and cells were counted using a hemocytometer. To isolate BEVs (Figure 1A), the *Bb*B31 culture was grown for 7–10 days in BSK-II supplemented with extracellular vesicle-free rabbit serum (serum was spun at 100,000× *g* for 1 h and verified by NTA to yield < 10 particles/mL). Spirochetes were pelleted at 5000× *g* for 15 min, followed by centrifugation to remove cellular debris at 10,000× *g* for 30 min, ensuring that the supernatant retained just BEVs. The supernatant was passed through a 0.22 µm filter to remove any remaining bacterial cells and debris. BEVs were obtained by high-speed ultracentrifugation at 100,000× *g* for 1 h. BEVs were washed twice with PBS and resuspended in a final volume of 100–200 µL. BEVs were characterized and counted using a ZetaView nanoparticle analyzer (Particle Metrix, Ammersee, Germany) and a ZetaSizer dynamic light scattering analyzer (Malvern Pananalytical, Malvern, UK).

### 2.2. BEV Transmission Electron Microscopy

Transmission electron microscopy images were acquired as described in Jung et al. [41], with some modifications. Briefly, the *Bb*B31 BEV pellet was fixed in 1 mL of 2.5% glutaraldehyde in 100 mM sodium cacodylate, pH 7 (cacodylate buffer), overnight at 4 °C. The fixed pellet was washed with cacodylate buffer, then post-fixed in 2% osmium tetroxide for 1 h at 4 °C. The pellet was washed with cacodylate buffer, dehydrated in a graded acetone series (50% to 100%), then embedded in Spurr’s Low Viscosity resin and cured at 65 °C for 18 h. Ultra-thin sections were mounted on 300 lacey-carbon-supported copper mesh TEM grids, post-stained with uranyl acetate and Reynolds’ lead citrate, and then imaged at 200 kV on a JEOL JEM-2100Plus TEM (JEOL, Tokyo, Japan).

### 2.3. Antibody Generation

The sequences ATAPSQGGVNSPVNV and GTGNRNQENDKDTPYNKT were used as the antigen for FlaB and p66 antibody generation, respectively. The production of monoclonal antibodies was contracted to Sino Biological. Briefly, Sino Biological synthesized and validated the antigens via UV analysis, immunized mice (N = 5) with the antigens (3 rounds of immunization), and performed serum titer testing. The mouse with the highest titer value was selected for B-cell isolation and subsequent hybridoma generation. At least 5 hybridomas per antigen were generated, and purified antibodies from each clone were evaluated via ELISA and Western blot analysis. Clones with the highest sensitivity via dot blot were considered for further analysis. The antigen peptides against which the antibodies were raised were also provided by Sino Biological. The anti-FlaB and p66 mouse monoclonal antibodies used for this study were GMU2-1-SM07 (SM07) and GMU2-3-SM10 (SM10), respectively.

### 2.4. Human Urine Samples

This study included 274 patients with acute Lyme disease (LD) and individuals with persistent symptoms following treatment for *borreliosis* [5] (Dr. Hope McIntyre, Lyme Hope LLC, MD). Acute LD patients had the characteristic erythema migrans (EM) rash and positive two-tier Lyme serology according to CDC criteria. Individuals with persistent symptoms after *borreliosis* treatment met the definition and grouping proposed by Fallon et al. [5]. The symptoms were judged by the physician to be functionally disabling. Symptoms included headaches, fatigue, brain fog, heart failure, dizziness, arthralgia, seizures, and vertigo (Table S1). Urine samples were collected either at the physician’s office or at the participants’ homes. Midstream urine was collected to reduce contamination. Urine samples were shipped to George Mason University at 4 °C overnight, stored at −80 °C upon arrival, and thawed only once [42]. The sample handling pipeline followed established guidelines for proteomics analysis [42].

### 2.5. Murine Urine Collection

A total of 25 six- to eight-week-old female C3H/HeJ mice were infected with *Borrelia burgdorferi* B31 either via intradermal needle injection [43,44,45] (N = 8 animals received 10^4^ spirochetes, N = 12 animals received 10^5^ spirochetes) or by exposure to approximately 15 infected nymphal ticks [46,47] per mouse (N = 5 animals). Five additional mice served as uninfected controls. Animals were sacrificed 7 and 28 days post-infection [44]. Before euthanasia, urine was collected by manual bladder expression (15–30 µL per mouse) and analyzed with proteomic mass spectrometry. The sample size (n = 25 infected; n = 5 uninfected controls) was selected for consistency with sample sizes commonly used in prior murine *Borrelia* infection studies and discovery proteomics workflows [48]. Group assignment was based on study logistics associated with infection route implementation and availability at each experimental time point. Urine samples were collected and processed in an interleaved order across groups to reduce batch effects. No animal or data point was excluded from the analysis. Scientists performing the proteomic analysis were not aware of group allocation. The primary outcome measure was the detection and characterization of *Borrelia*-derived proteins in urine, as assessed by proteomic mass spectrometry analysis. At sacrifice, mice were anesthetized with i.p. injection of ketamine (75 mg/kg)/medetomidine (1 mg/kg) and sacrificed by cervical dislocation.

### 2.6. Proteomic Mass Spectrometry Analysis

Human urine samples (40 mL minimum) were thawed overnight at 4 °C, analyzed using a Multistix 10 SG reagent strip (Siemens Healthineers AG, Forchheim, Germany), and centrifuged at 3700× *g* for 15 min to eliminate debris. After decanting, the pH was adjusted to 5.5 using 1 M hydrochloric acid or 1 M sodium hydroxide solutions. Urine samples were mixed with affinity hydrogel particles [48,49,50]. The affinity hydrogel particle–based pre-analytical workflow preserves target analytes from proteolytic degradation, enhances mass spectrometry sensitivity by more than 100-fold by excluding high-abundance background proteins, and maintains analytical linearity [51,52,53,54]. Urine samples (40 mL) were incubated with 200 µL of affinity hydrogel particle suspension (10 mg/mL) for 30 min at room temperature. The particles were separated by centrifugation at 19,000× *g* (Beckman Avanti JXN-26 Centrifuge, Beckman Coulter, Inc., Brea, CA, USA) for 45 min, and the supernatant was discarded. The particle pellet was washed twice by vigorous resuspension in 1 mL of 18 MΩ-cm water, followed by centrifugation at 16,100× *g* for 20 min. The supernatant was discarded, and the pellet was resuspended in 20 µL elution buffer solution (4% Rapigest, Waters, in 50 mM ammonium bicarbonate) and incubated for 20 min at room temperature. The samples were centrifuged at 16,100× *g* for 20 min. The eluates were reduced using 200 mM dithiothreitol at room temperature for 15 min and alkylated using 50 mM iodoacetamide at room temperature in the dark for 20 min. The trypsin digestion ran overnight with 2 µL of (0.5 µg/µL) of sequencing-grade trypsin (Promega, Madison, WI, USA, V5113) in 50 mM ammonium bicarbonate, pH 8, at 37 °C. A total of 2 µL of 100% trifluoroacetic acid (TFA) was added to the solution and allowed to incubate for 30 min. Digested samples were desalted using C-18 spin columns (Pierce, Appleton, WI, USA). The final eluates were dried using a nitrogen evaporator (Microvap 118, Organization Associates, Inc., Berlin, MA, USA). The samples were reconstituted in 10 µL 0.1% formic acid. Mouse urine samples were thawed, reduced, alkylated, trypsin digested, and analyzed individually by mass spectrometry without pre-analytical processing due to the small volumes. Liquid chromatography–tandem mass spectrometry (LC–MS/MS) was performed using an Orbitrap Fusion Tribrid Mass Spectrometer (Thermo Scientific, Waltham, MA, USA) coupled with a nano-spray EASY-nLC 1200 UHPLC. Reversed-phase chromatography separation of the peptide mixture was performed using a PepMap RSLC 75 μm i.d.  ×  15 cm long with a 2 μm C18 resin LC column (ThermoFisher, Waltham, MA, USA). Mobile phases A (0.1% formic acid) and B (0.1% formic acid and 80% acetonitrile) were used. Peptides were eluted using a linear gradient of 5% mobile phase B to 50% mobile phase B for 90 min at 300 nL/min and then to 100% mobile phase B for an additional 5 min. A Thermo Orbitrap Fusion Tribrid Mass Spectrometer (Thermo Scientific) was operated in data-dependent mode, in which each full MS scan was followed by TopN MS/MS scans of the most abundant molecular ions with charge states from 2+ to 4+, which were dynamically selected for collision-induced dissociation (CID) using a normalized collision energy of 35%. Tandem mass spectra were searched against *Borrelia* species (Table S2) and *Homo sapiens* NCBI and Uniprot databases using Proteome Discoverer 2.1 software using tryptic cleavage constraints. A peptide identification and authentication algorithm was used to ensure accurate peptide attribution to *Borrelia species*, with minimal false positives. **Peptide Spectrum Matching**: Stringent criteria included a peptide false discovery rate (FDR) of <1%; Xcorr values > 2.0, 3.0, and 4.0 for 2+, 3+, and 4+ precursor ions; q-value < 0.05; and precursor/fragment ion mass tolerances of <2 ppm and <0.5 Da. For triply charged ions, additional checks, such as the presence of basic residues and doubly charged precursor ions, were applied. **Microorganism Attribution**: Peptides were verified using the NCBI BLAST algorithm (https://blast.ncbi.nlm.nih.gov/Blast.cgi (accessed on 23 June 2025)) against the NCBI RefSeq database and the Uniprot BLAST algorithm (https://www.uniprot.org/blast (accessed on 23 June 2025)) against the UniProtKB reference proteomes+ Swiss-Prot. Peptides identical to sequences from non-target organisms were discarded, and those shorter than 7 amino acids were excluded to minimize random attributions.

### 2.7. Lipidomics Mass Spectrometry Analysis

MALDI-TOF MS analysis was performed using a PerSeptive BioSystems Voyager Elite DE-STR (Applied Biosystems, Foster City, CA, USA). Spectra were accumulated for 100 laser pulses at an attenuation of 2600. The instrument was operated in linear mode with a 20-kV accelerating voltage and a 150 ns ion extraction delay time.

### 2.8. Immunoblotting

Spirochetes were spun down at 5000× *g* and washed three times with PBS. Lysates were obtained by sonication of 500 µL of spirochete suspension for 15 cycles (10 s on and 15 s off) at a 30% power setting while keeping the sample on ice. Aliquots of 1 μL containing decreasing amounts of total protein (1.5, 0.7, 0.3 μg) were spotted on a nitrocellulose membrane and incubated overnight at 4 °C with primary antibodies (FITC-conjugated rabbit anti *B. burgdorferi*, 1:1000, Abcam; mouse anti-peptidoglycan monoclonal antibody, clone 4F7C4, 1:1000, Creative Diagnostics; *B. burgdorferi* OspA mouse monoclonal antibody, Clone ID: 5015, 1:500, Origene; mouse anti *B. burgdorferi* flagellar antigen, 1:1000, AbD Serotec/Bio-Rad (Hercules, CA, USA); mouse anti-*Borrelia* FlaB monoclonal antibody, 1:1000, clone GMU2-1-SM07; mouse anti-*Borrelia* p66 monoclonal antibody, 1:1000, clone GMU2-3-SM10). Signal was detected using appropriate horseradish peroxidase-conjugated goat anti-mouse or anti-rabbit antibodies (1:10,000, Abcam), the SuperSignal West Dura Extended Duration Substrate (ThermoFisher), and a ChemiDoc Imaging System (Bio-Rad). To isolate urinary extracellular vesicles (uEVs) from human urine samples, 10 mL of urine was centrifuged at 10,000× *g* for 30 min to remove cellular debris. The resulting supernatant was filtered through a 0.22 µm membrane to eliminate any remaining particulates. The filtrate was then subjected to high-speed ultracentrifugation at 100,000× *g* for 1 h. The resulting pellet was washed twice with PBS by centrifugation at 100,000× *g* for 1 h each and resuspended in a final volume of 100–200 µL. Aliquots of 1 µL of uEV suspension were manually spotted onto a nitrocellulose membrane and probed using mouse monoclonal antibodies against *Borrelia* FlaB (clone GMU2-1-SM07) and *Borrelia* p66 (clone GMU2-3-SM10), as described above.

### 2.9. HMC3 Microglia BEV Challenge

**Phagocytosis Assay**. In vitro phagocytosis in BbB31-BEV-treated and untreated HMC3 cells was detected by the Phagocytosis Assay Kit (Abcam, ab235901), which uses heat-killed, fluorescently pre-labeled *E. coli* particles as a tool for quantification of in vitro phagocytosis by fluorescent microscope, spectrophotometer, or flow cytometry (540/570 nm). BEV-treated HMC3 and untreated HMC3 cells were seeded into 96-well plates and incubated overnight to allow adherence and recovery. The following day, cell culture media were replaced, and cells were treated with effectors of interest or control media for one hour at 37 °C in a humidified 5% CO_2_ environment. Subsequently, 5 µL of Red *E. coli* suspension was added to each well, and the plate was incubated for an additional 2 to 3 h to facilitate bacterial uptake. After incubation, cells were harvested by centrifugation, washed multiple times with cold assay buffer containing effectors to remove non-internalized bacteria, and then resuspended for analysis. Phagocytosis was quantified by measuring the fluorescence intensity of internalized Red *E. coli* particles using flow cytometry, fluorescent microscopy, or a plate reader set to excitation/emission wavelengths of 540/570 nm. To correct for background fluorescence, readings from no-cell control wells were subtracted from all samples. A standard curve generated by serial dilution of Red *E. coli* was used to calibrate fluorescence measurements. A quenching solution was employed to distinguish internalized bacteria from those attached to the cell surface. The phagocytic response in experimental wells was expressed relative to positive controls to determine the percentage effect of various treatment conditions. In vivo phagocytosis in liposome (L-alpha phosphatidylcholine and cholesterol control, Ø = 100 nm, Creative Biolabs) and BEV-treated HMC3 cells was detected using Green Silica Fluorescent Particles, 100 nm, 10 mg/mL (Abvigen, Newark, NJ, USA). A total of 5 μL of particle suspension was directly added to cell culture media (10^7^ beads/mL/10^5^ cells) and incubated for 24 h. The FluoSpheres engulfment was visualized using a confocal microscope (Leica Microsystems, Wetzlar, Germany).

**Polymerase Chain Reaction (RT-qPCR).** BbB31-BEVs were treated prior to DNA extraction with a mild DNase digestion to remove externally associated DNA. Briefly, 200 µL of BEV suspension was incubated with DNase I (0.1 U/µL) at 37 °C for 20 min. The reaction was terminated by the addition of EDTA (10 mM final), and BEVs were subsequently purified by buffer exchange using Thermo Scientific Zeba Spin Desalting Columns to remove enzyme and digested nucleotides. DNA was isolated from 200 µL of purified BEV suspension (10^12^ particles) by phenol–chloroform extraction. An equal volume (200 µL) of phenol:chloroform:isoamyl alcohol (25:24:1, equilibrated to pH 8.0) was added to the BEV solution, mixed thoroughly, and centrifuged at 12,000× *g* for 10 min at 4 °C. The upper aqueous phase was transferred to a fresh tube, and DNA was precipitated with 0.1 volume of 3 M sodium acetate (pH 5.2) and 2.5 volumes of ice-cold 100% ethanol at –20 °C for 2 h. Samples were centrifuged at 12,000× *g* for 15 min at 4 °C, and the pellet was washed with 500 µL of 70% ethanol, air-dried, and resuspended in 50 µL nuclease-free water. DNA concentration as measured by NanoDrop was <0.1 ng/µL. PCR was performed using Q5 High-Fidelity DNA Polymerase (NEB) in 25 µL reactions containing 12.5 µL of 2× Q5 Master Mix, 0.5 µM of each primer (Table 1), 7 µL of DNA template, and nuclease-free water to volume. Cycling conditions were as follows: initial denaturation at 98 °C for 30 s; 30–35 cycles of 98 °C for 10 s, 58 °C for 20 s, and 72 °C for 30 s; final extension at 72 °C for 5 min; hold at 4 °C. PCR products were analyzed on a 1% agarose gel stained with SYBR Safe.

Total RNA was isolated from 500,000 HMC3 cells using Trizol (Invitrogen, Carlsbad, CA, USA). A 500 ng RNA template was used to generate cDNA (iScript™ Reverse Transcription Supermix for RT-qPCR, Bio-Rad, Hercules, CA, USA). The gene expressions were detected via standard RT-qPCR using specific primers (IDT, Coralville, IA, USA) listed in Table 2 and SYBR green master mix (Bio-Rad), which were performed on a thermal cycler (Thermo Scientific Applied Biosystems QuantStudio 7 Pro Real-Time PCR). The relative gene expression was quantified using the ΔΔCT method, normalized to GAPDH expression that was assayed with three technical replicates. The results of the triplicate experiments were depicted as mean ± standard error of the mean.

**Immunocytochemistry.** Cells grown on coverslips were washed with PBS and fixed in 4% paraformaldehyde in PBS for 15–20 min at room temperature. After fixation, the cells were washed and permeabilized with 0.1% Triton X-100 in PBS for 10 min at room temperature. Following three washes with PBS, blocking was performed with 5% BSA in PBS for 45 min to 1 h at room temperature. Coverslips were then incubated overnight at 4 °C with primary antibodies diluted in 5% BSA/PBS. The next day, cells were washed three times with 0.1% Triton X-100 in PBS (5 min each wash) and incubated with fluorescently labeled secondary antibodies diluted in 5% BSA/PBS for 1 h at room temperature in the dark. After incubation, cells were washed three times with 0.1% Triton X-100/PBS in the dark. Nuclear staining was performed using NucBlue/DAPI reagent for 5 min at room temperature, then washed once with PBS. Finally, coverslips were mounted with Prolong Gold Antifade medium, allowed to cure overnight at room temperature in the dark, and stored at 4 °C until imaging.

## 3. Results

### 3.1. Borrelia burgdorferi-Derived Bacterial Extracellular Vesicles (BEVs) Are Double-Membraned Particles ~150 nm in Diameter

In order to isolate BEVs, *B. burgdorferi* B31 (*Bb*B31) was grown in extracellular vesicle-free BSK-H or BSK-II medium at 37 °C for 10 days. BEVs were isolated using the procedure reported in Figure 1A, which included centrifugation, filtration, and ultracentrifugation. Fluorescence microscopy images of *B. burgdorferi* B31 stained with FITC-conjugated anti-*Borrelia* antibodies displayed distinct spirochete morphology (Figure 1B). *B. burgdorferi* B31 grown in BSK-II or BSK-H produced an average of 38,180 and 83 BEV particles per cell over 10 days, respectively, while the total BEV count in 10 mL of medium was 4.2 × 10^12^ and 8.5 × 10^9^, respectively (Table 3). For comparison, BEV counts for *B. afzelii*, *B. garinii*, *Borrelia miyamotoi*, and *B. hermsii* grown in BSK-H medium for ten days ranged from 1.2 × 10^9^ to 4.4 × 10^9^ (Table 3). The median diameter of *B. burgdorferi* B31 BEVs was 143.3 and 172.7 nm, when measured by dynamic light scattering (DLS) and ZetaView analysis, respectively (Figure 1C,D). Transmission electron microscopy (TEM) imaging of BbB31-BEVs at magnifications of 25,000×, 50,000×, and 100,000× confirmed their size range (Figure 1E). BEVs appeared as circular structures (~100–150 nm in diameter) with electron-dense borders and the discernible presence of a double-membrane structure (Figure 1E).

### 3.2. BEVs Contain Immunomodulatory Molecules Including Peptidoglycan, p66, and FlaB

**Figure 2:**
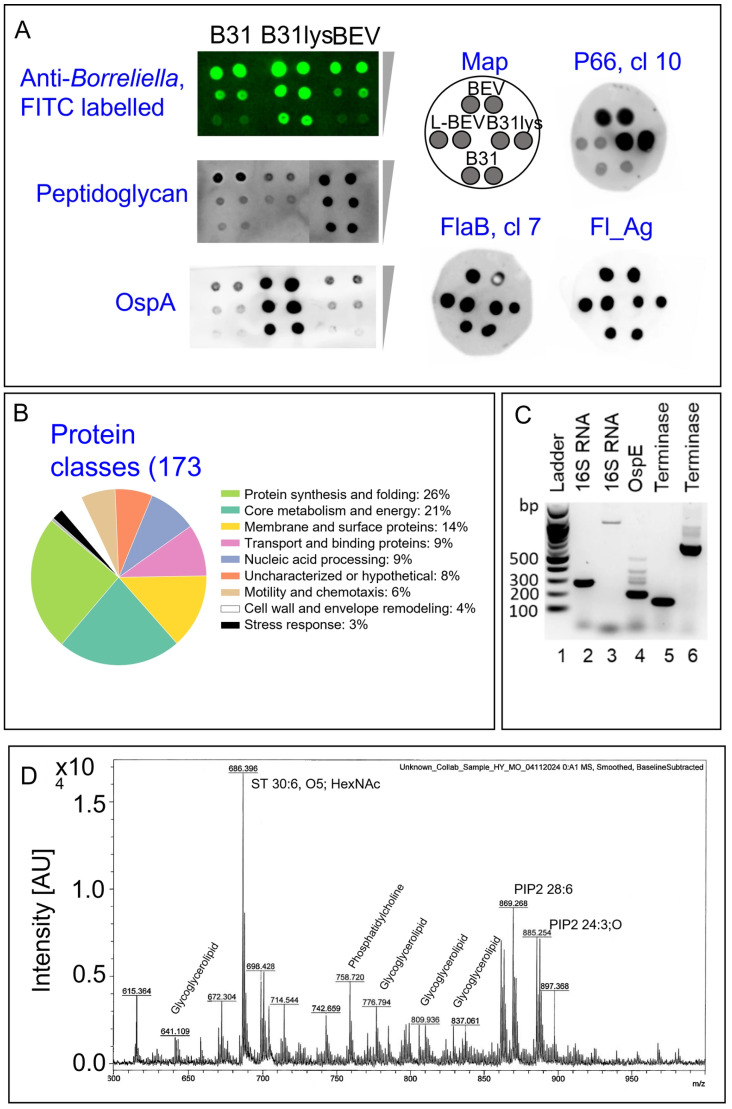
*Borrelia* BEVs contain immunomodulatory proteins, DNA, and lipids. (**A**) Dot blot analysis of intact *B. burgdorferi* B31 (B31), *Bb* B31 lysate (B31lys), intact BEVs (BEV), and lysed BEVs (L-BEV), probed with antibodies against *Borrelia* (FITC-labeled), peptidoglycan, OspA, p66 (SM10), FlaB (SM07), and Flagellar antigen (Fl_Ag), confirmed the presence of immunogenic proteins and cell wall components in BEVs. (**B**) Functional classification of 173 proteins identified in *Bb* BEVs by mass spectrometry shows representation across multiple biological processes, including protein synthesis and folding, core metabolism, membrane proteins, and nucleic acid processing. (**C**) BEV-associated DNA includes the chromosomal *16S rRNA* gene and cp32 plasmid-encoded *OspE* and *terminase* genes. Agarose gel electrophoresis of PCR products generated using the primer sets described in Table 1. Lane 1: molecular weight marker, Lane 2: *Bb 16S*-1-F/*Bb 16S*-1-R, Lane 3: *Bb 16S*-2-F/*Bb* *16S*-2-R, Lane 4: *Bb ospE*-78F/*Bb ospE*-282R, Lane 5: *Bb_Term1*-F/*Bb_Term1*-R, Lane 6: *Bb_Term2*-F/*Bb_Term2*-R. (**D**) MALDI-TOF mass spectrometry analysis of BEV lipid composition revealed peaks corresponding to glycosylated sterols, phosphatidylinositol 4,5-bisphosphate (PI(4,5)P_2_), ether-linked phosphatidylcholine, and glycosylglycerolipids.

In order to determine whether BbB31-BEVs contain immunomodulatory molecules, we performed a comprehensive molecular analysis at the protein, DNA, and lipid levels. Dot blot analysis with FITC-labeled anti-*Borreliella*, anti-peptidoglycan, anti-OspA, anti-p66, and anti-FlaB antibodies showed positive reactivity for intact and lysed *B. burgdorferi* B31 spirochetes and BEVs (Figure 2A). A mass spectrometry-based proteomic analysis identified a total of 173 proteins in the BEVs (Table S2). These Borrelia proteins have the following functions: protein synthesis and folding, core metabolism and energy, membrane and surface proteins, transport and binding, nucleic acid processing, motility and chemotaxis, and cell wall and envelope remodeling (Figure 2B).

Next, we performed PCR followed by agarose gel analysis, which demonstrated that BEVs contained both chromosomal and plasmid DNA, including the chromosomally encoded *16S* *rRNA* gene and the cp32 plasmid-encoded *OspE* and *terminase* genes (Figure 2C).

The lipid composition of BEVs was characterized using MALDI-TOF mass spectrometry [55]. The resulting spectrum displayed peaks compatible with glycosylated sterols (cholesteryl HexNAc-pentaol (30:6; O5), *m*/*z* 686.396 [M + H]^+^), phosphatidylinositol 4,5-bisphosphate (PI(4,5)P_2_ 38:4 *m*/*z* 885.254 [M − H]^−^, PI(4,5)P_2_ 36:4 *m*/*z* 869.268 [M − H]^−^), ether-linked phosphatidylcholine (PC(O-16:0/18:2), *m*/*z* 758.720), and glycosylglycerolipids (e.g., cholesteryl 6-O acyl beta D galactopyranoside *m*/*z* 809.936 [M + Na]^+^, 837.061 [M + Na]^+^) (Figure 2D).

### 3.3. BEV-Associated Proteins Are in the Mammalian Host Urine at Early Stages of Borreliosis and in Post-Treatment Symptomatic Patients

In order to collect data in support of a systemic presence of BEV markers in the mammalian host, we employed proteomic mass spectrometry and immunoassays to analyze *Borrelia*-infected mouse and human urine.

**Figure 3:**
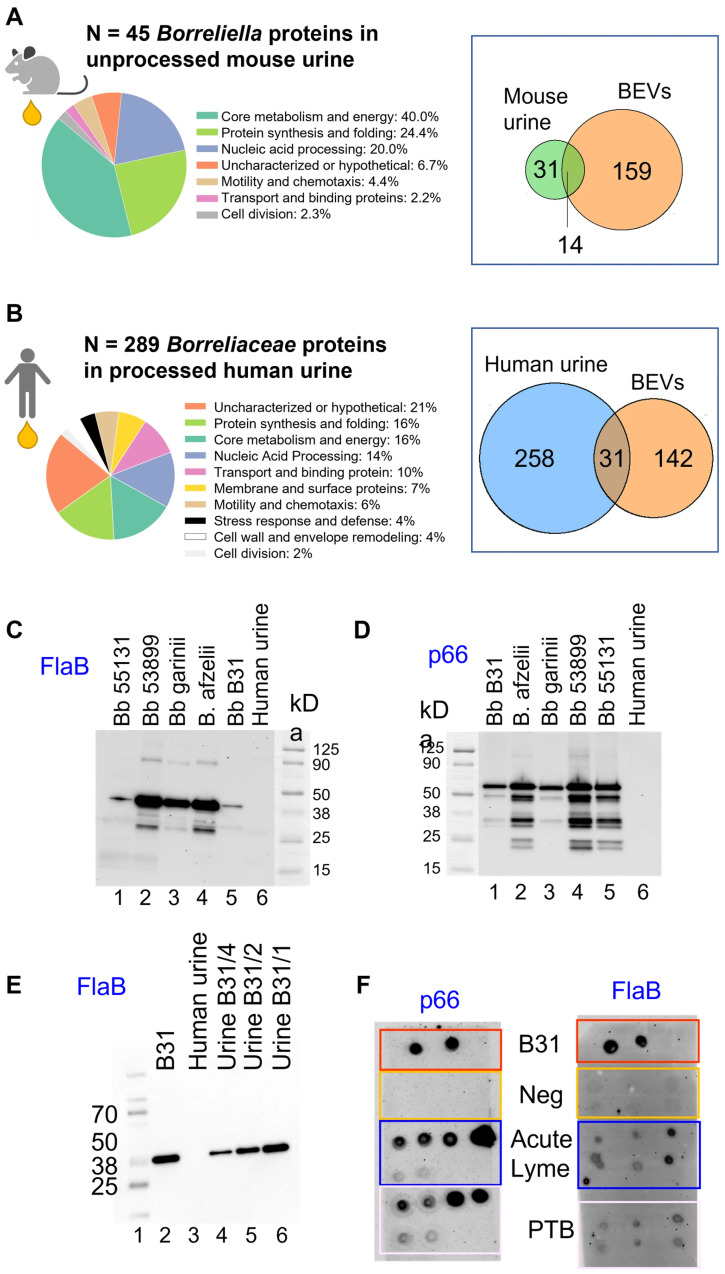
BEV-associated proteins are detected in mammalian urine during *Borrelia* infection and post-treatment symptom persistence. (**A**) Mass spectrometry analysis of urine from *Borrelia*-infected mice (n = 25) identified 45 *Borrelia*-derived proteins spanning metabolic, motility, and nucleic acid processing functions. A Venn diagram shows that 15 proteins overlap with those found in *B. burgdorferi* B31-derived BEVs. (**B**) Proteomic profiling of processed urine samples from 274 human participants revealed 289 *Borrelia* proteins, including 31 overlapping with the BEV proteome. Functional classification is shown as a pie chart. (**C**,**D**) Western blot validation of the anti-FlaB (**B**) and anti-p66 (**D**) antibodies shows cross-reactivity with multiple *Borrelia* species, including *B. burgdorferi* B31, 55131, and 53899, and *B. afzelii* and *B. garinii*. (**E**) Western blot validation of the specificity of the anti-FlaB antibody, showing dose-dependent detection of the *Bb* B31 lysate spiked in human urine, and no reactivity in urine alone. Lane 1: molecular weight ladder; lane 2: *Bb* B31 lysate (1.4 µg); lane 3: human urine; lane 4: *Bb* B31 lysate spiked in urine 0.34 µg; lane 5: *Bb* B31 lysate spiked in urine 0.7 µg; lane 6: *Bb* B31 lysate spiked in urine 1.4 µg. Uncropped images for (**C**–**E**) are available in Supplementary Figure S1. (**F**) Dot blot analysis of urinary extracellular vesicles (uEVs) from representative patients with acute Lyme disease and with persistent symptoms following treatment for *borreliosis* (PTB), reveals detection of p66 and FlaB in clinical specimens, supporting systemic dissemination of vesicle-associated cargo and the feasibility of marker detection.

To determine whether *Borrelia*-derived peptides could be detected in mammalian urine under conditions of unequivocal spirochetal infection, we employed a murine model. A total of 25 C3H/HeJ mice were infected with *Bb*B31—either via intradermal needle injection or by exposure to approximately 15 infected nymphal ticks per mouse. Five additional mice served as uninfected controls. In total, 34 peptides with shared identity to *B. burgdorferi* and other bacterial species were cross-detected in uninfected urine and removed from the study (Table S2B). Urine was collected from each mouse (15–30 µL) at 7 and 28 days post-infection [44] and analyzed individually by mass spectrometry without pre-analytical processing due to the small sample volumes. Despite these sensitivity constraints, urine samples from infected mice yielded 45 *Borrelia* proteins (Table S2) spanning several functional categories, including core metabolism and energy production (e.g., pyruvate kinase, pyrophosphate–fructose 6-phosphate 1-phosphotransferase), protein synthesis (e.g., 30S ribosomal proteins, translation initiation factor IF-2), nucleic acid processing (e.g., DNA primase, DNA gyrase), and motility and chemotaxis (e.g., chemotaxis protein CheA, flagellar biosynthesis protein FlhA) (Figure 3A). In the absence of a pre-analytical processing step, membrane and surface proteins, as well as proteins involved in cell wall and envelope remodeling, were not detected. Fourteen of the proteins identified in mouse urine overlapped with those found in *B. burgdorferi* B31-derived BEVs (Table 4). These findings confirm the detectability of *Borrelia* proteins in infected mammalian urine and support the hypothesis that BEVs contribute to the amplification, systemic dissemination, and urinary excretion of *Borrelia*-derived peptides.

We conducted a mass spectrometry-based discovery proteomics analysis on urine samples collected from 274 individuals (age average = 41 ± 22 years, 39% males, Table S1) with either acute Lyme disease or persistent symptoms following treatment for *borreliosis* [5]. From these samples, we identified a total of 289 unique *Borrelia* species-derived proteins (Figure 3B, Table S2). Higher participant numbers, larger sample volumes, and affinity purification may have contributed to more robust detection in human compared to mouse urine samples. The largest functional group was protein synthesis and folding (45 proteins), accompanied by a high number of uncharacterized or hypothetical proteins (61), indicating both active protein processing engagement and the presence of poorly understood or novel factors. A substantial portion of the proteins were associated with core metabolism and energy (45), nucleic acid processing (28), such as enzymes for DNA replication and transcription, and transport and binding proteins (35), many of which are likely involved in nutrient acquisition and host interaction. Membrane and surface proteins (21) included immunogenic components potentially involved in immune evasion and pathogenesis. Other functional categories included proteins involved in motility and chemotaxis (17), stress response and defense (13), cell wall and envelope remodeling (12), and cell division (7). To explore the potential contribution of BEVs to urinary protein shedding, we compared this dataset to the 173 BEV proteins. This comparison revealed an intersection of 31 proteins (Figure 3B, Table 4). The intersecting proteins spanned several key functional categories, including membrane-associated components (e.g., outer membrane protein p66), proteins involved in protein synthesis and folding (e.g., 30S ribosomal proteins, chaperone protein DnaK), and enzymes central to metabolic pathways (e.g., glyceraldehyde-3-phosphate dehydrogenase, glycerol kinase). The presence of these diverse proteins in both BEVs and human urine supports the hypothesis that BEVs are shed during infection, circulate systemically, and that their molecular cargo is ultimately expelled in urine. To verify the proteomics findings, we developed targeted immunoassays.

From the subset of BEV-associated proteins detected in human urine, we prioritized p66 and FlaB for further validation based on their essential roles in membrane adhesion/transport and motility, respectively, as well as their well-documented immunogenicity across multiple *Borrelia* species. To account for the genetic heterogeneity of *Borrelia* species, we generated anti-FlaB and anti-p66 antibodies against epitopes predicted in silico to be conserved across multiple Lyme disease–associated *Borrelia* species, including *B. burgdorferi sensu stricto*, *B. afzelii*, *B. garinii*, *B. bissettii*, and *B. mayonii*. The cross-reactivity of these antibodies was experimentally validated against several strains of *B. burgdorferi* (B31, 55131, 53899), *B. afzelii,* and *B. garinii* (Figure 3C,D). Antibody specificity was confirmed using human urine as a background matrix, into which serial dilutions of *B. burgdorferi* B31 lysate were spiked. The detection of a clear, dose-dependent signal accounting for over 95% of total signal intensity and the absence of reactivity in urine alone, demonstrated high specificity (Figure 3E for FlaB; Datla et al. [56] for p66). Using these antibodies and the extracellular vesicle isolation procedure described in Figure 1A, we detected both FlaB and p66 in urinary extracellular vesicles (uEVs) from representative patients with acute Lyme disease and those experiencing persistent symptoms following *borreliosis* treatment [5] (Figure 3F). These findings provide in vivo evidence for urinary excretion of BEV-associated components.

### 3.4. BEV Challenge Altered Microglia Immunometabolic and Inflammatory Responses

In order to investigate whether *Borrelia* BEVs were capable of immune cell activation and modulation, we treated HMC3 microglial cells with *in* vitro-generated BEVs at different time points and recorded data on HMC3 phagocytic activity and expression of genes and proteins involved in the inflammatory, immunometabolic, and antioxidant responses.

**Figure 4:**
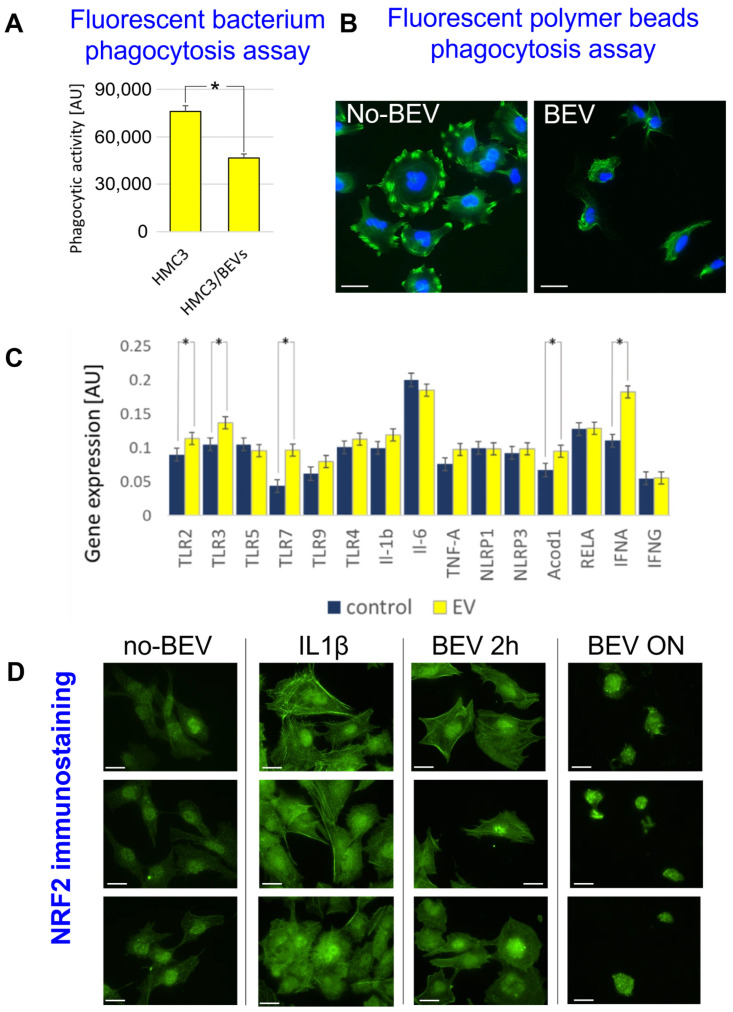
*Borrelia* BEVs impair phagocytosis and activate inflammatory and antioxidant pathways. (**A**) Quantification of HMC3 phagocytic activity using the Abcam Red *E. coli* phagocytosis assay kit shows a significant fluorescence decrease upon BEV exposure compared to untreated controls (*t*-test *p* = 0.02). HMC3 = untreated cells, HMC3/BEV = BEV-treated cells. (**B**) Representative fluorescence images of the phagocytosis assay with FluoSpheres beads confirm reduced bead internalization in BEV-treated cells. No-BEV = liposome treatment, BEV = BEV treatment. (**C**) Gene expression profiling revealed that BEVs significantly upregulated transcripts associated with innate immune activation (e.g., TLR2, TLR3, TLR7, INFα, Acod1), indicating a pro-inflammatory signature. (**D**) Immunostaining for NRF2 demonstrates time-dependent nuclear translocation in HMC3 cells treated with BEVs (2 h and overnight), surpassing the effect of IL-1β. No-BEV = liposome treatment, IL-1β = IL-1β treatment, BEV 2h = 2 h BEV treatment, BEV ON = overnight BEV treatment. * *p* < 0.05 (Student’s *t*-test). Scale bar = 30 µm.

The phagocytic activity of HMC3 cells was assessed using the Vybrant™ Phagocytosis Assay Kit by monitoring the internalization of a foreign particle, fluorescein-labeled non-viable *Escherichia coli* (K-12 strain) cells. Quantitative analysis revealed a significant reduction in phagocytic efficiency in BEV-treated HMC3 cells (100 particles/cell, overnight) compared to untreated controls (fold change = 0.6, *t*-test *p* value = 0.02, Figure 4A). Green silica fluorescent particles were used in an uptake assay to confirm these results. As depicted in Figure 4B, cells exposed to BEVs (100 particles/cell, overnight) showed a marked decrease in particle uptake compared to liposome-treated controls, confirming impaired phagocytic function in response to BEVs.

Gene expression analysis of BEV-treated HMC3 cells (100 particles/cell, overnight) identified a distinct pro-inflammatory and immunometabolic signature (Figure 4C). Genes encoding Toll-like receptors (TLR2, TLR3, TLR7), Acod1, and interferon alpha (INFα) showed significantly increased expression (* *p* < 0.05), while the TLR5, TLR9, TLR4, IL1β, IL-6, TNF-α, NLRP1, NLRP3, and RELA mRNA abundance was unaffected by BEV treatment. This data supports that BEVs induce a pro-inflammatory response in HMC3 cells.

Finally, to evaluate whether BEVs activate antioxidant and stress response pathways in HMC3 cells, we conducted immunofluorescence analysis of NRF2 nuclear translocation. BEV-treated HMC3 cells (100 particles/cell for 2 h and overnight, BEV panels) exhibited a time-dependent increase in NRF2 nuclear localization compared to liposome-treated cells (no-BEV panel), with complete translocation observed following overnight exposure. The effect of BEVs exceeded that of IL-1β (5 ng/mL overnight), a known inducer of NRF2 nuclear translocation [57].

These findings support a model in which BEVs drive HMC3 activation, alter immunometabolic reprogramming, and impair phagocytic function.

## 4. Discussion

Human data from acute Lyme disease (LD) suggest a paradox: despite the low abundance of *Borrelia* organisms and the absence of known toxins, the host mounts a robust inflammatory response [8]. For individuals with prolonged symptoms, immunologic studies show that immune responses can become dysregulated via persistent inflammation, potential autoimmunity, or nonspecific immune activation [8]. Involvement of the peripheral and central nervous system (CNS) has also been implicated, with evidence from human and animal studies showing activation of microglia and monocytes in perivascular spaces contiguous with the cerebrospinal fluid (CSF), along with elevated CSF cytokines in spirochetal CNS infections [44]. Mechanisms by which non-neurological infections impact neurobehavioral functions include the diffusion of bacterial exotoxins or peripheral cytokines into the CNS [58]. The initiating trigger of immune dysregulation remains uncertain and may occur independently of ongoing infection [8]. The presence of persistent antigenic debris, containing peptidoglycan, in antibiotic-refractory Lyme arthritis is widely recognized [10,59]. However, the structural composition of this antigenic debris in the mammalian host remains incompletely defined [60]. This gap in mechanistic understanding is mirrored by the absence of validated diagnostics or FDA-approved treatments for persistent post-treatment Lyme disease symptoms.

**Figure 5:**
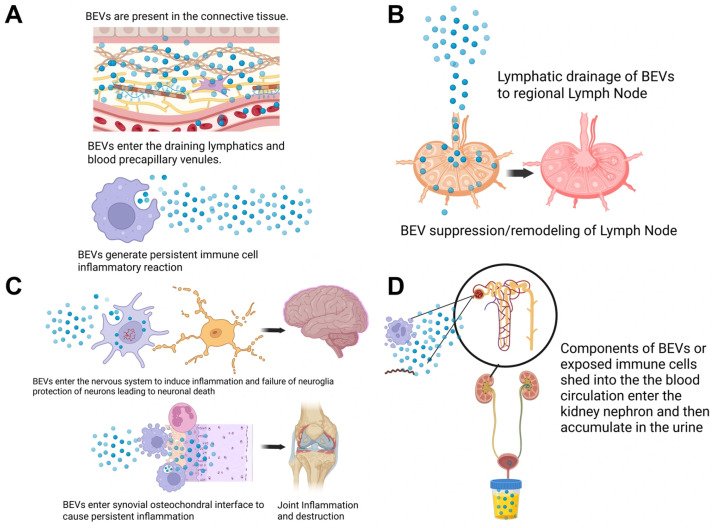
Working model of bacterial extracellular vesicle (BEV) dissemination, immunopathology, and BEV component urinary excretion. (**A**) Gram-negative BEVs exist in the interstitium, enter draining lymphatics and blood precapillary venules, and persist at tissue sites, where they sustain localized immune activation even in the absence of a replicating bacterium. (**B**) BEVs traffic to regional lymph nodes via lymphatic drainage, where they may contribute to immune suppression and structural remodeling of lymphoid tissue. (**C**) BEVs disseminate systemically and may reach the nervous system to activate microglia and promote neuronal injury or localize to synovial joints where they trigger chronic inflammation and tissue destruction. (**D**) BEVs, either directly released by *bacteria* or from exposed host cells, circulate through the bloodstream, and BEV components are filtered by the kidney nephron, resulting in their accumulation and detection in urine.

We propose a working model in which *Borrelia* bacterial extracellular vesicles (BEVs) disseminate systemically via lymphatic and vascular routes and contribute to immunopathological processes (Figure 5). Although this model remains theoretical for *Borrelia*, it is supported by biodistribution studies of BEVs derived from other Gram-negative bacteria [61,62]. BEVs have been shown to access the interstitium, draining lymphatics, and blood microvasculature [63,64,65], suggesting a potential route for dissemination. Following lymphatic transport, BEVs reach regional lymph nodes, where they may contribute to lymph node suppression or remodeling, potentially disrupting normal immune function [64,65]. Circulating BEVs can also access the nervous system and interact with resident immune and neural cells, promoting neuroinflammation and impairing neuroglial protection, potentially leading to neuronal damage [66,67]. Microvesicles can accumulate in joint synovial tissue, particularly at the osteochondral interface, where they sustain chronic inflammation and contribute to joint damage and destruction [68,69]. Finally, BEV markers can reach the kidney nephron via systemic circulation and are ultimately excreted in the urine [70]. This evidence supports the potential of BEV-associated cargo as a noninvasive biomarker of infection.

Rich in antigenic and immunomodulatory components, BEVs might have the potential to disrupt immune responses and damage host tissues, even in the absence of viable bacteria at those sites. This is supported by literature showing that microbiome-derived BEVs can enter circulation and access distant organs, including the brain [61,71]. In tick-borne infection models such as tick-borne encephalitis and Langat virus, extracellular vesicles served as mediators of CNS inflammation [72]. In Alzheimer’s disease, BEVs from *Paenalcaligenes hominis*, a Gram-negative gut bacterium, modulated microglial activation via TLR4 and the NLRP3 inflammasome, leading to cytokine production (IL-1β, IL-6, TNF-α) and neurotoxicity [73]. In mice, these BEVs induced cognitive deficits and increased microglial activation in the hippocampus [73]. In Gram-negative bacteria, BEVs typically form through outer membrane blebbing due to cell envelope biosynthesis imbalances or incorporation of hydrophobic molecules [74,75]. An alternate mechanism, explosive cell lysis, has been shown in *Pseudomonas aeruginosa*, where endolysins encoded by cryptic phage regions degrade peptidoglycan, fragment membranes, and form BEVs [75]. BEVs range from 10 to 300 nm and may possess single or double bilayer membranes that retain bacterial surface proteins [76,77] (Figure 1E and Figure 2A). In *Borrelia*, BEV production has been observed near division sites and across the outer membrane [22], both in culture [23] and in tick midguts during early feeding [24,25]. Unlike other bacteria, *Borrelia* demonstrates limited protein secretion capacity [23], reinforcing a possible role of BEVs in delivering effector molecules.

In this study, BEV production per spirochete varied depending on the growth medium. Higher *B. burgdorferi* BEV yield in BSK-II compared to BSK-H may reflect differences in lipid availability. The relatively lower lipid concentration in BSK-II may promote membrane remodeling to meet metabolic demands, leading to membrane destabilization that enhances vesicle release. Genotype-dependent variation in outer membrane architecture and lipid metabolism [78] may also explain strain-specific differences in BEV output [79]. To exclude medium-derived particle contamination, both media were ultracentrifuged at 100,000× *g* prior to use and analyzed by nanoparticle tracking analysis (NTA), confirming the absence of detectable BEV-sized particles.

We report for the first time the presence of *Borrelia* peptidoglycan, a known immunogen in antibiotic-refractory Lyme arthritis [10,59], within intact BEVs, supporting the hypothesis that BEVs act as stable carriers of antigenic material. We also identified the glycolipid BbGL-I (cholesteryl 6-O-acyl-β-D-galactopyranoside), previously shown to be immunogenic in mice [55]. It has been proposed that BEVs, coated with such molecules, could bind antibodies during their generation, potentially dampening their microbicidal activity [80]. BEV-mediated lipid transfer to host cells may influence immune recognition and antigen presentation [25,81].

BEVs also carried high molecular weight DNA (*16SrRNA*), *OspE*, and *terminase* genes) [82] (Figure 2C). This may reflect incorporation of chromosomal or plasmid fragments during vesicle biogenesis, particularly given the segmented linear and circular plasmid architecture of Borrelia [83]. Vesicle-associated DNA has been reported in multiple Gram-negative species [84,85,86] and may contribute to innate immune activation via TLR9 or cGAS–STING pathways [87,88] or potentially facilitate horizontal gene transfer [89].

Proteomic analysis of *Borrelia* BEVs (Table S2) revealed the presence of several highly immunogenic cell wall-associated proteins that elicit antibody responses in humans: basic membrane proteins A/B/D, BdrV, GroEL, CRASP-1, ErpA8, FlaB, glycerophosphodiester phosphodiesterase, p37 (Uniprot ID O50846), p66, OMS28, p13, OspA/B/C, and VlsE [25]. The less-characterized outer membrane protein BBA03 [90] may also play a role in immune interactions. BEVs also carried proteins that are known to influence host–pathogen interactions. For example, plasminogen-binding proteins (e.g., OspC, GroEL, and enolase) promote extracellular matrix degradation [91,92,93]; CRASP-1 and ErpA mediate complement evasion by binding to Factor H, FHL-1, CFHR1/2, and components C7/C9, thus preventing membrane attack complex formation [94]. Other proteins, such as BmpA/B/D, BdrV, CRASP-1, p66, OspC, and VlsE, directly interact with host extracellular matrix and receptors [25,95,96,97,98]. However, it is unclear how these proteins may function and impact the host in these non-replicating reservoirs. Intracellular bacterial proteins identified in BEVs (Table S2), including ribosomal proteins, aminopeptidases, DNA/RNA processing enzymes, and redox enzymes such as L-lactate dehydrogenase, thioredoxin, and superoxide dismutase, may also retain enzymatic activity [99], affecting host cell behavior and immune signaling.

We identified 14 and 31 *Borrelia* BEV proteins in murine and human urine, respectively, supporting systemic dissemination and highlighting their potential as noninvasive biomarkers. Urine was selected as the biofluid of choice because it has demonstrated utility for detecting infections originating outside the urinary tract and contains proteins derived from multiple organ systems, including the liver, brain, lung, skin, heart, intestine, and pancreas [49,50,100,101,102,103]. The protein repertoire identified in in vitro–generated BEVs did not fully overlap with that detected in mammalian urine (Figure 3), potentially reflecting host adaptation processes occurring in vivo. Further supporting a role for the host environment in shaping *Borrelia* physiology and vesicle cargo, only 12.5% of BEV proteins detected in mammalian urine were shared between murine and human hosts (Table 4).

We generated two mouse monoclonal antibodies against conserved antigens of FlaB (ATAPSQGGVNSPVNV) and p66 (GTGNRNQENDKDTPYNKT), unique to disease-causing *Borrelia* species and not shared with other organisms (NCBI non-redundant database). Western blot analysis confirmed cross-reactivity across multiple *Borrelia* species and demonstrated high specificity, with no detectable reactivity against human or commensal microbial proteins naturally present in urine. These antibodies were able to detect both intact and lysed *B. burgdorferi* B31 spirochetes and BEVs. We detected BEV-associated proteins in acute Lyme patients and individuals with *borreliosis* post-treatment symptoms 0–24 months after diagnosis.

BEVs from various bacterial species can alter macrophage responses, modulate antigen presentation, and trigger programmed cell death [104]. In our BEV challenge experiments, *Borrelia* BEVs caused microglial dysregulation in HMC3 cells: upregulation of IFN-α and Acod1, Nrf2 nuclear translocation, and reduced phagocytic activity. IFN-α enhances microglial activation but, when dysregulated, contributes to neuroinflammation. Elevated IFN-α correlates with neurocognitive symptoms in Lyme patients [105]. Acod1, upregulated in macrophages upon *Borrelia* exposure [39], mediates immunometabolic shifts and can contribute to neurodegeneration and immune paralysis when aberrantly expressed [40]. Based on prior literature, a plausible mechanism explaining how *Borrelia* BEVs impair microglial phagocytosis is that BEV-associated p66 engages SIRPα and, by functionally mimicking CD47, suppresses phagocytic engulfment [106]. The presence of OspC may also impair phagocytosis [107]. Independent in vitro and co-culture models demonstrated that microglia exposed to *Borrelia* adopt a pro-inflammatory M1-like phenotype [108] and can mediate bystander neuronal apoptosis through inflammatory signaling, even in the absence of direct bacterial effects on neurons [109]. Importantly, a murine study of neuroborreliosis showed that acetate supplementation attenuated CNS inflammation [110]. As acetate is converted to acetyl-CoA and enters the TCA cycle, it may counteract the glycolytic shift associated with inflammatory activation and promote mitochondrial metabolism [110]. Collectively, this evidence supports the concept that microglial metabolic reprogramming contributes to disease pathogenesis and highlights microglia-driven neuroinflammation as a potentially actionable therapeutic target. Accumulating evidence indicates that not only live *Borrelia* but also bacterial debris can robustly activate primary human microglia, inducing pro-inflammatory mediators and neurotrophic factors that may initiate and sustain neuroinflammation [111].

This study has several limitations. *B. burgdorferi* strain B31 was cultured in both BSK-H and BSK-II media to evaluate medium-specific effects on BEV production and to assess variability in spirochete growth and vesicle release associated with differences in nutrient composition. The comparative analysis was limited to strain B31, which served as the reference strain throughout this study. Strain- and media-dependent differences in BEV production will be examined in future investigations focused on genotype-specific vesicle biogenesis and cargo composition. Further studies are needed to investigate the physiological drivers of *Borrelia* BEV production and whether the production rate and BEV composition depend on environmental conditions (e.g., tissue type, immune cell interaction, oxidative stress) or the *Borrelia* growth phase. Future work is warranted to expand the investigation of BEV-induced microglia activation, including protein-level verification of the gene expression analysis, uptake mechanisms, and downstream signaling characterization, such as bactericidal, antigen-presenting, and cell-death pathways. HMC3 cells were used as a well-characterized, immortalized human microglial model that retains key phagocytic and inflammatory functions, enabling reproducible in vitro assays. However, microglial responses are likely patient-specific and influenced by receptor expression, genetic and epigenetic background, and baseline inflammatory state, warranting validation in primary or patient-derived models. Ex vivo studies were limited to using in vitro-generated BEVs. In vivo-generated BEVs might have somewhat different antigenic compositions that cannot currently be replicated using in vitro culture. In vivo studies on BEV biodistribution, persistence, behavior upon antibiotic treatment, and in vitro analyses with other cell types (e.g., astrocytes, neurons, synoviocytes, macrophages) and human tissue biopsies will provide critical insights.

## 5. Conclusions

In conclusion, *Borrelia* BEVs contain DNA, immunogenic and metabolic proteins, and membrane components such as peptidoglycan and lipids. Two novel monoclonal antibodies against conserved p66 and FlaB epitopes successfully detected *Borrelia* proteins in infected mammalian urine. Overlap between *Borrelia* BEV and urinary proteins supports systemic dissemination of vesicle-associated components and the feasibility of their detection as biomarkers. BEVs altered microglial function in vitro, supporting a mechanism of immune dysregulation that may contribute to persistent inflammation. These findings support the role of *Borrelia* BEVs in Lyme disease pathogenesis and their potential utility as diagnostic and therapeutic targets.

## Figures and Tables

**Table 1:** PCR primers used for the amplification and detection of *Borrelia* genes.

Gene	Primers
Bb ospE-78F	TGATGGGCAAAGTAATGGAGAGG
Bb ospE-282R	AAAGAATGTAGCGGTGTATCCTGC
Bb 16S-1-F	GGCCCGAGAACGTATTCACC
Bb 16S-1-R	CGAGCGCAACCCTTGTTATC
Bb 16S-2-F	AGAGTTTGATCATGGCTCAG
Bb 16S-2-R	GGTTACCTTGTTACGACTT
Bb_Term1-F	GAGTGGATAGCAAGCACTGAT
Bb_Term1-R	ATCATCAACTCGCTCCATAACA
Bb_Term2-F	GGAGGCATAGCTAGTGGCAAA
Bb_Term2-R	CCGCCAACACTAAATGCT

**Table 2:** PCR primers used for the amplification and detection of human genes.

Gene	NCBI Accession Number	Primers
*hIFN-α Interferon alpha 1*	3439	5′-GACTCCATCTTGGCTGTGA 3′-TGATTTCTGCTCTGACAACCT
*hIFNG Interferon gamma*	3458	5′-GTATTGCTTTGCGTTGGACA 3′-GAGTGTGGAGACCATCAAGGA
*TNF-α* *Tumor necrosis factor*	7124	5′-GTCAACCTCCTCTCTGCCAT 3′-CCAAAGTAGACCTGCCCAGA
*IL-1B Interleukin 1 beta*	3553	5′-GGAGAATGACCTGAGCACCT 3′-GGAGGTGGAGAGCTTTCAGT
*IL6 Interleukin 6*	3569	5′-AGTCCTGATCCAGTTCCTGC 3′-CTACATTTGCCGAAGAGCCC
*RELA NF-κB p65 subunit*	5970	5′-CTACGACCTGAATGCTGTGC 3′-CTGCCAGAGTTTCGGTTCAC
*NLRP1 NLR family pyrin domain containing 1*	22,861	5′-TCCCCCTTGGGAGTCCTCCTGAAAATG 3′-CGAGAACAGCTGGTCTTCTCCAGGGCTTC
*NLRP3 NLR family pyrin domain containing 3*	114,548	5′-CTTCTCTGATGAGGCCCAAG 3′-GCAGCAAACTGGAAAGGAAG
*TLR2 Toll-like receptor 2*	7097	5′-TGATGCTGCCATTCTCATTC 3′-CGCAGCTCTCAGATTTACCC
*TLR3 Toll-like receptor 3*	7098	5′-GTATTGCCTGGTTTGTTAATTGG 3′-AAGAGTTCAAAGGGGGCACT
*TLR4 Toll-like receptor 4*	7099	5′- AAGCCGAAAGGTGATTGTTG 3′-CTGAGCAGGGTCTTCTCCAC
*TLR5 Toll-like receptor 5*	7100	5′-GGAACCAGCTCCTAGCTCCT 3′-AAGAGGGAAACCCCAGAGAA
*TLR7 Toll-like receptor 7*	51,284	5′-CCTTGAGGCCAACAACATCT 3′-GTAGGGACGGCTGTGACATT
*TLR9 Toll-like receptor 9*	54,106	5′-CAGCAGCTCTGCAGTACGTC 3′-AAGGCCAGGTAATTGTCACG
*Acod1 Aconitate decarboxylase 1*	730,249	5′-TTCCATGAATGCCAGATCAA 3′-GGTTTTCTCCAGTGCCCATA

**Table 3:** Spirochete seed number, spirochete harvest number at 10 days, number of BEV particles isolated from cultures grown in BSK-H and BSK-II medium, and ratio of BEV particles per spirochete cell.

Spirochete Species	Seed Number	Harvest Number	Number of BEVs	BEV per Spirochete
*B. burgdorferi* (BSK-H)	10^6^	1.02 × 10^8^	8.5 × 10^9^	83
*B. hermsii* (BSK-H)	10^6^	1.57 × 10^8^	1.2 × 10^9^	8
*B. afzelii* (BSK-H)	10^6^	1.20 × 10^8^	4.4 × 10^9^	37
*B. garinii* (BSK-H)	10^6^	1.15 × 10^8^	1.9 × 10^9^	16
*B. miyamotoi* (BSK-H)	10^6^	1.61 × 10^8^	1.5 × 10^9^	9
*B. burgdorferi* (BSK-II)	10^6^	1.10 × 10^8^	4.2 × 10^12^	38,182

**Table 4:** Proteins common to mammalian host fluids and in vitro-grown *Borrelia* BEVs, with reference to Figure 3A,B.

Set	Protein Name	Uniprot ID	Ordered Locus ID
Mouse/BEV intersection	2,3-bisphosphoglycerate-dependent phosphoglycerate mutase	O51602	BB_0658
	Chemotaxis protein CheA	O51515	BB_0567
	DNA gyrase	O51396	BB_0435
	DNA topoisomerase 4	O51066	BB_0035
	Glucose-6-phosphate isomerase	O51672	BB_0730
	Lon protease 1	Q59185	BB_0253
	Lysine–tRNA ligase	O51603	BB_0659
	Polyribonucleotide nucleotidyltransferase	O51745	BB_0805
	Protein-glutamate methylesterase/protein-glutamine glutaminase	Q45047	BB_0568
	Pyrophosphate–fructose 6-phosphate 1-phosphotransferase	P70826	BB_0020
	Pyruvate kinase	O51323	BB_0348
	Tyrosine–tRNA ligase	O51343	BB_0370
	Uncharacterized lipoprotein BBD10	P70837	BB_D10
	V-type ATP synthase	O51120	BB_0093
Human/BEV intersection	30S ribosomal protein S13	O51453	BB_0500
	Aminopeptidase II	O51096	BB_0069
	Basic membrane protein A	Q45010	BB_0383
	Chaperone protein DnaK	P0C922	BB_0518
	Chemotaxis protein CheA	O51292	BB_0312
	DNA gyrase	O51396	BB_0435
	Elongation factor G	O30913	BB_0540
	Flagellar protein FliL	Q57442	BB_0279
	Flagellin FlaB	P11089	BB_0147
	Glyceraldehyde-3-phosphate dehydrogenase	P46795	BB_0057
	Glycerol kinase	O51257	BB_0241
	Glycerol-3-phosphate dehydrogenase	O51259	BB_0243
	Integral outer membrane protein p66	H7C7N8	BB_0603
	L-lactate dehydrogenase	O51114	BB_0087
	Methyl-accepting chemotaxis protein	O51542	BB_0596
	Outer surface protein B	P17739	BB_A16
	PF-49 plasmid partition protein	H7C7N6	BB_P33
	Phosphoglycerate kinase	Q59181	BB_0056
	Protein translocase subunit SecA	O07497	BB_0154
	Pyrophosphate–fructose 6-phosphate 1-phosphotransferase	P70826	BB_0020
	Pyruvate kinase	O51323	BB_0348
	Septation protein SpoVG	O51726	BB_0785
	Thioredoxin	O51088	BB_0061
	Transcription termination/antitermination protein NusG	O51355	BB_0394
	Trigger factor	O51555	BB_0610
	Triosephosphate isomerase	Q59182	BB_0055
	tRNA nucleotidyltransferase (CCA-adding enzyme)	O51649	BB_0706
	V-type ATP synthase	O51120	BB_0093
	recombinase RecA	Q59180	BB_0131
	chaperone protein GrpE	P28609	BB_0519
	Protein Smf	Q44773	BB_0297

## Data Availability

The authors declare that all data supporting the findings of this study are available within the article and its Supplementary Information Files. Raw files for LC-MS/MS protein analysis will be deposited in the MassIVE database (https://massive.ucsd.edu/ProteoSAFe/static/massive.jsp, accessed on 23 June 2025) under accession codes PDX074641, PDX074644, PDX074645, and PDX074646.

## Supplementary Materials

The following supporting information can be downloaded at https://www.mdpi.com/article/10.3390/microorganisms14030600/s1, Table S1: Demographic and clinical information of the human participants who donated urine under informed consent. Participants included patients with acute Lyme disease and individuals with persistent symptoms following treatment for borreliosis. Table S2: List of the Borrelia proteins identified in *B. burgdorferi* B31 bacterial extracellular vesicles (BEVs), mouse, and human urine. Proteins are involved in core metabolism and energy, protein synthesis and folding, membrane and surface proteins, transport and binding, nucleic acid processing, motility and chemotaxis, and cell wall and envelope remodeling. Supplementary Figure S1. Uncropped Western blot images relative to Figure 3.
