# Supplementary material for: Characterization of Borrelia-Derived Extracellular Vesicles: Implications for Pathogenesis and Diagnostics
Source: Microorganisms. 2026 Mar 7;14(3):600. doi: 10.3390/microorganisms14030600 (PMC13029535; doi:10.3390/microorganisms14030600)
Supplement: Supplementary file 1 [file microorganisms-14-00600-s001.zip › Supplementary Figure 1.pptx]

## Slide 1
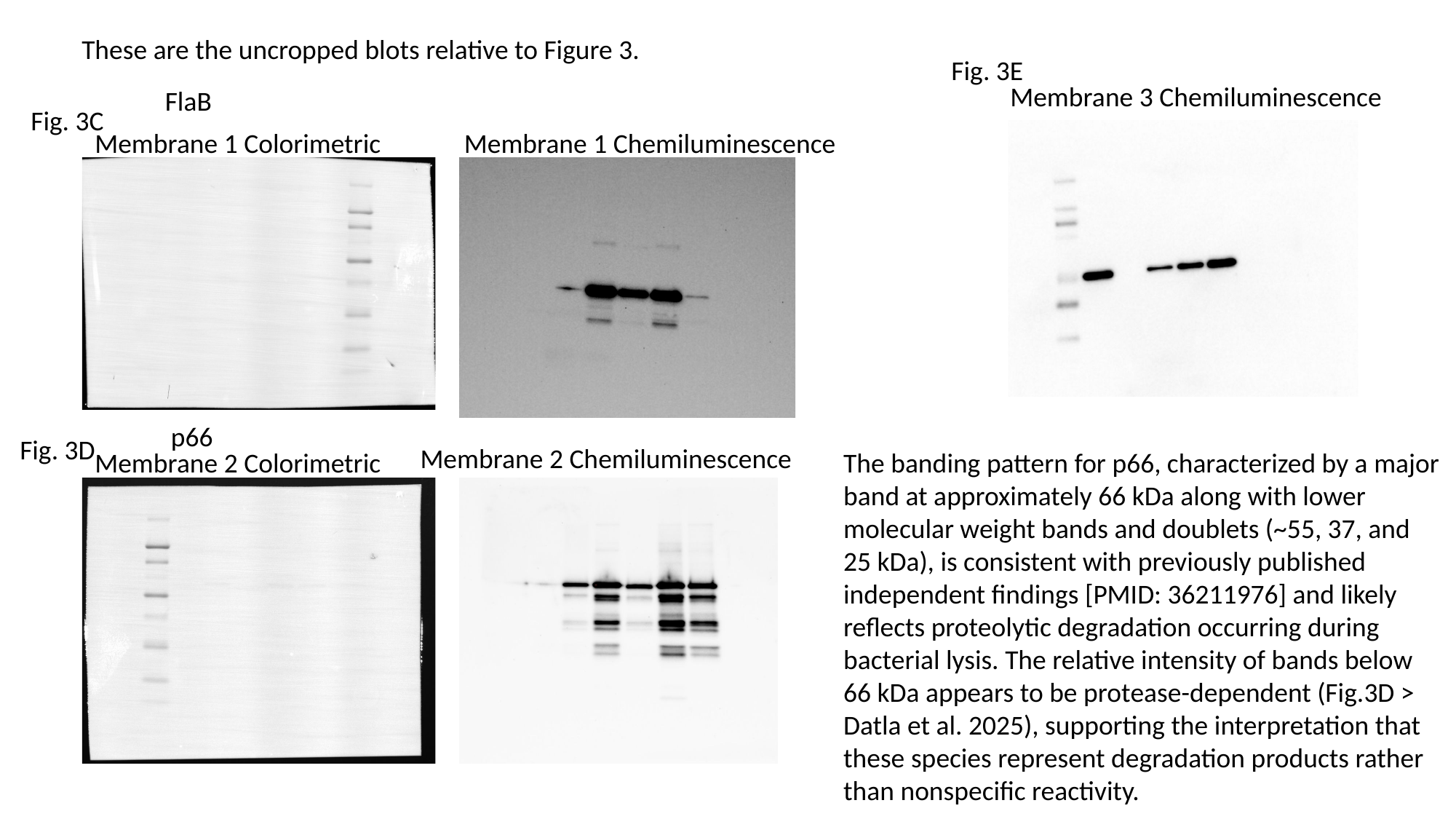

These are the uncropped blots relative to Figure 3.
Fig. 3E
Membrane 3 Chemiluminescence
FlaB
Fig. 3C
Membrane 1 Colorimetric
Membrane 1 Chemiluminescence
p66
Fig. 3D
Membrane 2 Chemiluminescence
Membrane 2 Colorimetric
The banding pattern for p66, characterized by a major band at approximately 66 kDa along with lower molecular weight bands and doublets (~55, 37, and 25 kDa), is consistent with previously published independent findings [PMID: 36211976] and likely reflects proteolytic degradation occurring during bacterial lysis. The relative intensity of bands below 66 kDa appears to be protease-dependent (Fig.3D > Datla et al. 2025), supporting the interpretation that these species represent degradation products rather than nonspecific reactivity.
